# Transcriptome Sequencing Reveals Regulatory Mechanisms of Taxol Synthesis in *Taxus wallichiana* var. *Mairei*

**DOI:** 10.1155/2019/1596895

**Published:** 2019-05-02

**Authors:** Tao Wang, Yiming Chen, Weibing Zhuang, Fengjiao Zhang, Xiaochun Shu, Zhong Wang, Qing Yang

**Affiliations:** ^1^Jiangsu Key Laboratory for the Research and Utilization of Plant Resources, Institute of Botany, Jiangsu Province and Chinese Academy of Sciences (Nanjing Botanical Garden Mem. Sun Yat-Sen), Nanjing, Jiangsu 210014, China; ^2^College of Life Sciences, Nanjing Agricultural University, Nanjing, Jiangsu 210095, China

## Abstract

Taxol is one of the most potent and effective anticancer drugs and is originally isolated from *Taxus* species. To investigate the specific regulatory mechanisms of taxol synthesis in *Taxus wallichiana* var. *mairei*, RNA-seq was conducted to reveal the differences in transcriptional levels between wild type (WT) and “Jinxishan” (JXS), a cultivar selected from a population of *Taxus mairei* that shows about 3-fold higher taxol content in the needles than WT. Our results indicated that high expressions of the genes taxadienol acetyltransferase (*TAT*), taxadiene 5-alpha hydroxylase (*T5H*), 5-alpha-taxadienol-10-beta-hydroxylase (*T10OH*), and 2-debenzoyl-7,13-diacetylbaccatin III-2-O-benzoyl-transferase (*DBBT*), which catalyze a series of key acetylation and hydroxylation steps, are the main cause of high taxol content in JXS. Moreover, in the present study, the activation of jasmonic acid (JA) signal transduction and its crosstalk with gibberellin (GA), auxin, and ethylene (ET) explained the elevation of differentially expressed genes (DEGs) from the taxol biosynthesis pathway. This also indicates that taxol biosynthesis in *T. mairei* is associated with the balance of cell development and defense. TF-encoding (transcriptional factor) genes, represented by the ethylene-responsive transcription factor (ERF), basic/helix-loop-helix (bHLH), MYB, and WRKY families, were detected as differentially expressed between JXS and WT, further indicating that the regulation of hormone signaling on taxol biosynthesis genes was mediated by transcription factors (TFs). To our knowledge, this is the first study to illustrate the regulatory mechanisms of taxol synthesis in a new cultivar of *T. mairei* with a high taxol content in its needles. These transcriptome data provide reasonable explanations for the variation of taxol content between WT and JXS.

## 1. Introduction

Taxol, originally isolated from the bark of *Taxus brevifolia* [1], is one of the most effective antitumor drugs for the treatment of several cancers, such as breast, lung, and ovarian cancers [2]. With the increasing incidence of cancer, the commercial value of taxol has grown prominently. However, due to limited resources and low productivity of *Taxus* species, the production of taxol is not sufficient to meet market demands [3].

Great efforts have been made to increase taxol production. In addition to screening for *Taxus* species with high levels of taxol [4], several alternative methods have been explored, such as total synthesis of taxol [5], plant cell culture [6], and taxol-producing fungi [7]. However, most of these methods are difficult to scale because of the large quantity of organic solvents consumed and low efficiency. Therefore, for the foreseeable future, *Taxus* species will remain a source for taxol and related precursors. In this regard, the supply of taxol and its precursors will undoubtedly depend on understanding the taxol biosynthesis pathway.

The biosynthesis pathway of taxol has been basically elucidated, and it involves 19 steps of enzymatic reaction [8]. It is generally believed that the pathway can be divided into three main stages. The first stage is the formation of a taxane skeleton, which mainly concerns the cyclization of a geranygeranyl skeleton to form taxadiene under the catalysis of taxadiene synthase (*TS*). Second, taxadiene goes through a series of reactions, including hydroxylation, acylation on hydroxyl groups, ketolation, and the formation of epoxypropane to form baccatin III, one of the major substrates for the chemical semisynthesis of taxol. The reaction process in this stage is complicated and requires multiple enzymes to cocatalyze the reaction step by step [9]. Lastly, the assembly of a C13-side chain attached to baccatin III is thought to be the final step of the pathway [10]. Since Croteau et al. [2] demonstrated the pathway of taxol biosynthesis, the key enzyme genes related to taxol biosynthesis and the relationship between the expression levels of these genes and the synthesis of taxanes have been extensively studied. Ajikumar et al. [11] have successfully overexpressed genes, including *TS* and *T5H* in *Escherichia coli*, and promoted the synthesis of taxodiene to about 1 g·L^−1^. Zhou et al. [12] have cocultured yeast containing *T5H* and its reductase gene cytochrome P450 reductase and *E. coli* containing *TS* that produces 33 mg·L^−1^ of oxygenated taxanes. The continued improvement in the efficiency of taxol synthesis will undoubtedly depend on a comprehensive understanding of its biosynthesis pathway, especially the enzymes that catalyze each step and their encoding genes.

RNA-seq, a highly accurate and cost-effective DNA sequencing technology, was developed as a powerful tool to analyze the functional complexity of transcriptomes for nonmodel organisms without a reference genome [13, 14]. In addition, RNA-seq can detect very low-level transcripts and provide information on the transcriptional structure and gene expression profiles [15, 16]. Currently, RNA-seq has been applied to investigate various aspects of taxol biosynthesis in different *Taxus* species. Hao et al. [17] reported the tissue-specific transcriptome of *T. mairei* using Illumina sequencing and analyzed the expression levels of genes related to taxol biosynthesis in three different tissues (root, stem, and leaves). Yu et al. [18] investigated the differences between *Taxus media* and *T. mairei* at the transcriptional level and suggested that the variation in taxoid content may be attributed to the differential expression of candidate genes involved in taxoid biosynthetic pathways. The early response of elicitation with methyl jasmonic acid (MeJA) in *T. chinensis* cells was studied by Li et al. [3], who found that a series of TFs, such as MYB, bHLH, ERF, AP2, and MYC, activated by exogenous MeJA may be involved in the regulation of gene expression in the taxol synthesis pathway. Deep sequencing of *T. media* cells has revealed an important role for miRNA in the regulation of gene expression associated with terpenoid backbone and paclitaxel biosynthesis following induction by MeJA [19]. However, a comprehensive understanding of the regulation of gene expression profiles in response to taxol biosynthesis is still lacking, which may be due to the lack of genomic information.

*Taxus wallichiana* var. *mairei*, a member of the Taxaceae, is a tall evergreen tree mainly distributed in southeastern China [20]. *T. mairei* is a unique and endangered species in China that is widely used for ornamental, material, and medicinal purposes [21, 22], and it is considered a major source for the cost-effective production of taxane drugs [17]. The color of the aril in *Taxus* species is usually red, but yellow has been reported in the arils of *Taxus Lutea* [23]. In 2015, a cultivar with yellow arils was found in a population of *T. mairei*, which was located in Wuxi, Jiangsu Province, China. Based on the results of internal transcribed spacer sequence analysis, this cultivar, named Jinxishan (JXS), was a natural mutant of *T. mairei.* Interestingly, the average content of taxol in the needles of JXS was determined to 1.7-fold higher than that in the wild type (WT) [24], and some superior individual trees of JXS showed even more accumulation of taxol in the needles, making them an ideal source for investigating the mechanisms of taxol biosynthesis. Therefore, superior individual trees of JXS and WT were analyzed by RNA-seq to describe the transcriptome and reveal transcriptome profiles in the present study. This is the first study to illustrate the taxol synthesis pathway based on the transcriptome profile of a new cultivar of *T. mairei* with a high taxol content in the needles. Our results provide insight into the regulatory pattern and network formation in the biosynthesis of taxol in *Taxus* species.

## 2. Materials and Methods

### 2.1. Plants of WT and JXS

Plants of WT and JXS were cultivated in a germplasm nursery for *Taxus* species located in Wuxi, Jiangsu Province, China (120°32′E, 31°43′N). For the cultivating stage, individuals of JXS and WT at the same age were cultivated closely and under the same conditions, such as sunshine and water. Needles of triplicate samples were collected from 12-year-old WT and JXS plants in March, June, September, and December of 2017, respectively, for taxol determination. Moreover, needles of WT and JXS were collected in June independently for RNA extraction.

### 2.2. Taxol Determination

Needles collected from WT and JXS plants were dried at 65°C for about 6 h and powdered. Five grams of powder was weighed with high precision and added to 100 mL, 50 mL, and 50 mL methanol in turn for methanol reflux extraction, according to the method of Li et al. [25]. The supernatants of the 3 extractions were combined and condensed to 100 mL. Then, the concentrates were extracted with N-hexane in equal volume for 4 times. The extracts were dried by rotary evaporation, and methanol was added to dilute the products to 25 mL. Before HPLC analysis, the samples were filtered through 0.22 *μ*m membrane filters.

The quantification of taxol was carried out using Agilent-1100 high-performance liquid chromatography and an Agilent DAD monitor. The separation of taxol was achieved on a Curosil-PFP C_18_ column of 250 mm × 4.6 mm and 5 *μ*m particle size. The mobile phase composed of acetonitrile and water was gradient eluted at a flow rate of 2.6 mL/min under 30°C, and the injection volume was 10 *μ*L [25]. A standard solution of taxol was employed to create a standard curve for quantification.

### 2.3. RNA Extraction

Total RNAs were isolated by a plant RNA kit (Omega, Norcross, USA) according to the manufacturer's protocol. DNA contamination was removed during the RNA extraction process. The RNA quality was verified by RNase-free agarose gel electrophoresis and a 2100 Bioanalyzer (Agilent Technologies, Santa Clara, CA). High-quality RNA samples of the triplicates of JXS and WT were mixed in equal quantity for cDNA library construction and subsequent RNA sequencing. Six mixed RNA samples from needles of JXS and WT were finally obtained.

### 2.4. Library Construction and Sequencing

After total RNA was extracted, eukaryotic mRNA was enriched by oligo (dT) beads. Then, the enriched mRNA was fragmented into short fragments using fragmentation buffer and reverse transcripted into cDNA with random primers. Second-strand cDNAs were synthesized by DNA polymerase I, RNase H, dNTP, and buffer. Then, the cDNA fragments were purified with a QiAquick PCR extraction kit, end repaired, poly(A) added, and ligated to Illumina sequencing adapters. The ligation products were size selected by agarose gel electrophoresis, PCR amplified, and sequenced using an Illumina HiSeqTM 4000 by Gene Denovo Biotechnology Co. (Guangzhou, China).

### 2.5. *De Novo* Assembly and Read Annotation

Raw reads contain adapters or low-quality bases that could affect subsequent assembly and analysis. Thus, to get high quality clean reads, raw reads containing adapters, reads with more than 10% unknown nucleotides, and low-quality reads with over 40% low *Q*-value (≤20) bases were removed by the Perl program (version 5.18.4). Clean reads of six RNA samples were merged and *de novo* assembled using Trinity Package 2.0 to construct unique consensus sequences as a transcriptome reference. The unigene annotation was used with the BLASTx program (https://www.ncbi.nlm.nih.gov/BLAST/) with an *E*-value threshold of 10^−5^ for the NCBI nonredundant protein (Nr) database (https://www.ncbi.nlm.nih.gov), the Swiss-Prot protein database (https://www.expasy.ch/sprot), the Kyoto Encyclopedia of Genes and Genomes (KEGG) database (https://www.genome.jp/kegg), the (COG/KOG) database (https://www.ncbi.nlm.nih.gov/COG), and Gene Ontology (GO) classifications by Blast2GO (https://www.blast2go.com/). Protein functional annotations were then obtained according to the best alignment results.

### 2.6. Identification of DEGs

The clean reads were mapped to the reference transcriptome using Bowtie2 by default parameters, and the mapping ratio was calculated through the following equation: mapping ratio = (unique mapped reads number + multiple mapped reads number) / all reads number. The gene abundances were calculated and normalized to reads per kb per million reads (RPKM). Datasets of three distinct biological samples from WT and JXS were treated as a group, and the differential expression between the two groups was analyzed using edge R package (https://www.r-project.org/). Significant DEGs were identified with a fold change ≥ 2 and a false discovery rate (FDR) < 0.05. DEGs were then subjected to enrichment analysis of GO functions and KEGG pathways. First, these DEGs were mapped to GO (http://www.geneontology.org/) terms. The *p* values were adjusted with the FDR correction, and a corrected *p* value ≤ 0.05 was used for significantly enriched GO terms in DEGs. Moreover, for KEGG enrichment analysis, pathways with an FDR value ≤ 0.05 were recognized as enriched. The formula was the same as that used for GO analysis.

### 2.7. Real-Time PCR Validation

Quantitative real-time PCR (qRT-PCR) was conducted in an optical 96-well plate with an ABI7500 system (ABI, USA) and commercial SYBR® Premix Ex Taq II (Tli RNaseH Plus; TAKARA, Shanghai, China), using the same cDNA samples as used for the RNA-seq experiment.  Table S1 shows the primers for the selected genes and the reference gene 18S. Real-time PCR was carried out in a final volume of 20 *μ*L, which contained 1 *μ*L of cDNA. The PCR program was set as follows: initial denaturation at 95°C for 30 s, 40 cycles of denaturation at 95°C for 5 s, and annealing and extension at 60°C for 34 s. A melting curve was obtained at 95°C for 15 s and at 60°C for 1 min followed by continuous heating. Two independent biological replicates and three technical replicates for each PCR reaction were performed. Data analysis was performed with the REST 2009 software.

### 2.8. Statistical Analyses

Statistical analyses were conducted using the SPSS software version 19.0, and one-way ANOVA was applied to compare taxol content differences between JXS and WT.

## 3. Results

### 3.1. Differences in the Taxol Content between WT and JXS

**Figure 1:**
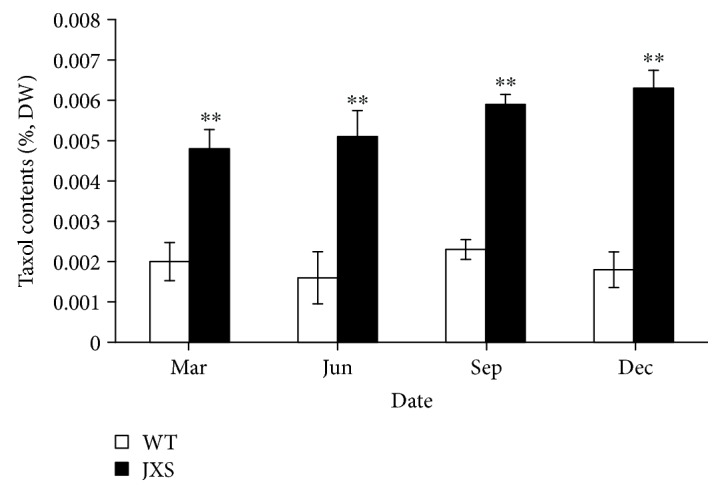
The paclitaxel contents (%, DW) in the needles of WT and JXS samples. Significant variations (*p* < 0.01) are indicated by ∗∗. Error bars represent means ± SD (*n* = 6).

The taxol contents in the needles of the collected samples are shown in Figure 1. The taxol contents in JXS were significantly higher than that of WT in the different months of the year (*p* < 0.01). Among them, the mean value of taxol in JXS was 0.0051% in June, while the mean value in WT was only 0.0016%. The average content of taxol in JXS was about 3.2-fold higher than that in WT, and this was statistically significant (*p* < 0.01).

### 3.2. Illumina Sequencing, Sequence Assembly, and Read Annotation

**Figure 2:**
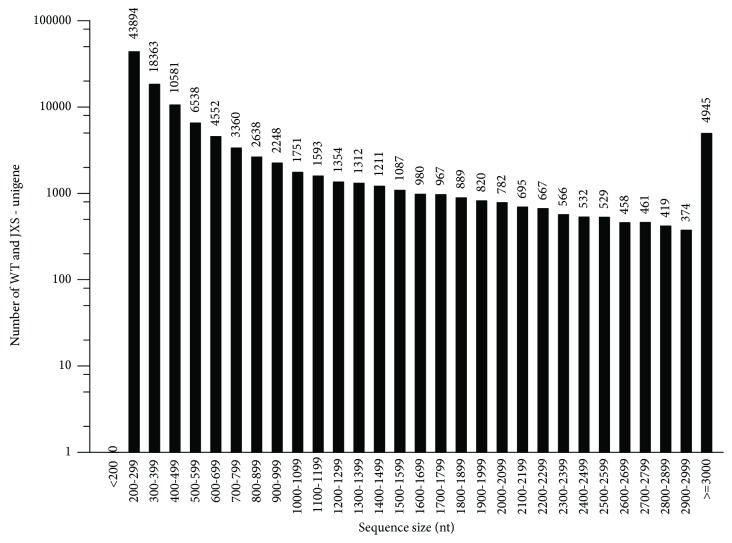
The length distribution of all assembled unigenes.

Illumina high-throughput second generation sequencing was used to obtain transcriptome data after total RNA was, respectively, extracted from the needles of WT (accession number: SRR8648837) and JXS (accession number: SRR8648838). In total, 55,961,361 and 62,206,034 high-quality reads of 150 bp sequences were generated from WT and JXS, respectively, after removing the adaptor sequences, empty reads, and low-quality reads. The Q20 percentage, N percentage, and GC percentage in WT and JXS were 98.96% and 98.61%, 0.02% and 0.01%, and 45.19% and 45%, respectively. All of the reads were assembled into 114,566 unigenes with a mean length of 761 bp and N50 size of 1,484 bp, using the Trinity software. The sequences of the unigenes were list in S2. The size distribution for these unigenes is shown in Figure 2. To gain preliminary insight into the functions of these unigenes, we performed a BLASTx search against the GenBank nonredundant protein database (Nr) with an *E*-value of 10^−5^ as a cutoff, and 38,310 unigenes (33.44% of the total) were annotated as Nr. Moreover, there were 26,823 (23.41%) matching protein sequences in the SwissProt database, 13,568 (11.84%) in the KEGG database, and 24,473 (21.36%) in the KOG database (Table S3). A number of *Taxus* unigenes showed high similarity to genes in other plant species. The largest number of *Taxus* homologous genes was identified in *Amborella trichopoda*.

**Figure 3:**
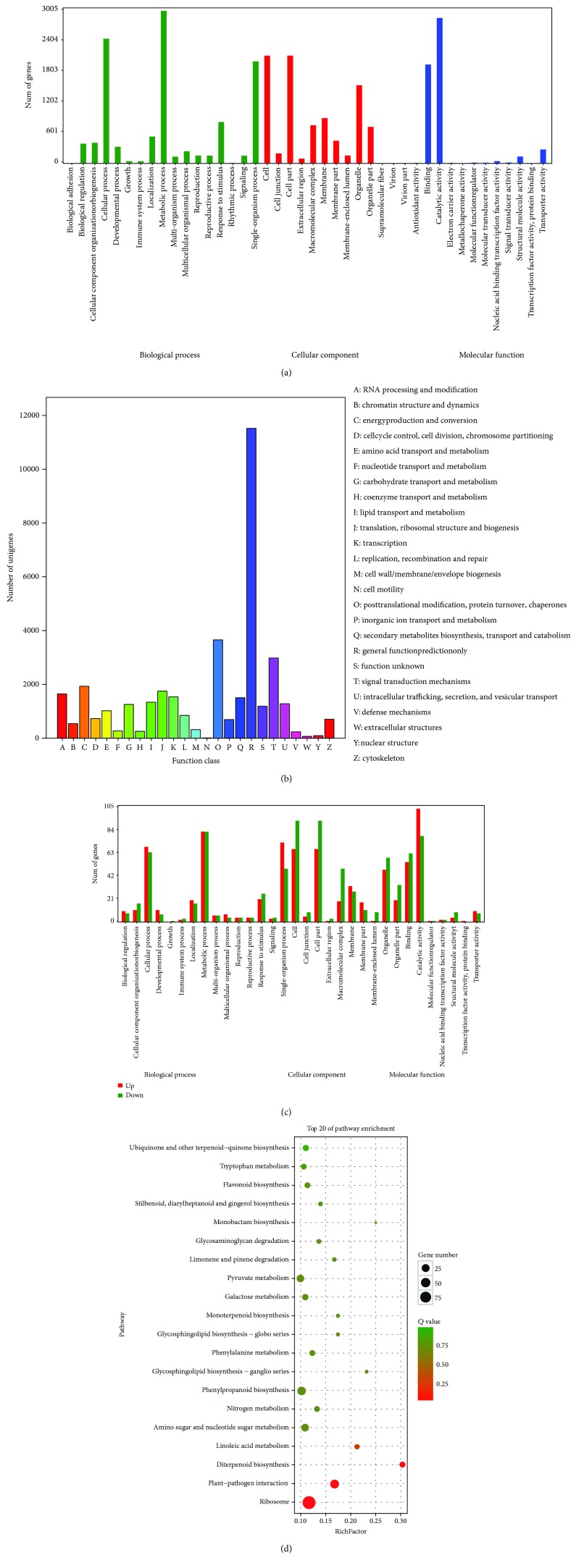
(a) GO annotation of all unigenes. Annotated sequences were classified into Šbiological process,” Šmolecular function,” and Šcellular component” groups and 42 subgroups. (b) KOG category classification of all unigenes. (c) GO classification of DEGs. (d) KEGG pathway enrichments of DEGs in TOP20.

GO terms were used to classify the functions of predicted unigenes. There were 5,290 out of 38,310 unigenes that were annotated with Blast2GO and were categorized into 42 functional groups in the three categories of molecular function, cellular components, and biological processes by the WEGO software. Among them, the seven GO terms of “metabolic process,” “catalytic activity,” “cellular process,” “cell,” “cell part,” “single-organism process,” and “binding” are presented in Figure 3(a). To further facilitate the functional classification of the unique sequences, the COG database was used to evaluate the integrality of the transcriptome library. In total, 24,473 out of 38,310 unigenes were divided into 25 different COG categories, which are represented by A to Z (Figure 3(b)). Among them, the cluster for the R category “general function prediction only” was the largest group, followed by the O category “posttranslational modification, protein turnover, chaperones” and the T category “signal transduction mechanisms.” Moreover, to understand the metabolic pathways in WT and JXS, a total of 7,810 annotated unigenes were assigned to 133 KEGG canonical pathways. Among them, the three most represented pathways were metabolic pathways (43.8%), biosynthesis of secondary metabolites (25.33%), and biosynthesis of antibiotics (12.57%) (Table S4).

### 3.3. GO and KEGG Enrichment Analysis of DEGs

**Figure 4:**
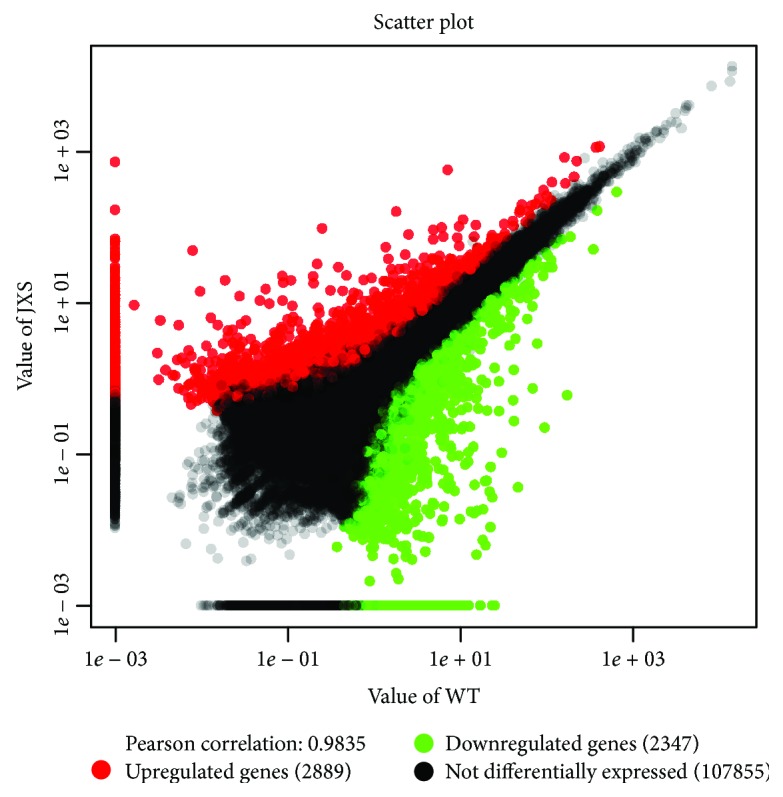
Expression levels in JXS vs. WT. The red points represent upregulated unigenes, the green points represent downregulated unigenes, and the black points represent non-DEGs.

The normalized expression value of genes was calculated by the RPKM method, and DEGs were identified and analyzed using the FDR method. A total of 5,236 prominently expressed unigenes were identified from the needles of WT and JXS (Figure 4). Compared with WT, the expression levels of 2,889 DEGs were upregulated and those of 2,347 DEGs were downregulated in JXS.

Most of the 5,236 DEGs between WT and JXS were significantly enriched in 6 GO terms. The most highly represented terms in the biological processes, cellular component, and molecular function category were “metabolic process” and “cellular process,” “cell part” and “organelle,” and “catalytic activity” and “binding,” respectively (Figure 3(c)). KEGG classifications were performed for a preliminary understanding of the reason for high taxol content in JXS compared to WT needles. DEGs enriched by KEGG were mainly involved in metabolic pathways, biosynthesis of secondary metabolites (diterpenoid biosynthesis and phenylpropanoid biosynthesis), translation, and environmental adaptation (*p* < 0.05) (Figure 3(d)). Apart from the pathways mentioned above, there were also a number of DEGs participating in signal transduction, such as plant hormone signal transduction.

### 3.4. DEGs Involved in Paclitaxel Biosynthesis

Taxol biosynthesis, a crucial part of diterpenoid biosynthesis, was one of the most concerned pathways in the present study. However, the taxol biosynthesis pathway was still incomplete, and only 11 genes were confirmed in the KEGG database. To further analyze how these DEGs contribute to the higher taxol in JXS, DEGs involved in the taxol biosynthesis pathway were identified by a reciprocal BLAST search against the transcriptome using previously reported enzymes as queries. Our transcriptome data revealed 109 genes involved in the taxol biosynthesis pathway (Table S5), which were assigned to 11 functional genes, and 6 genes were differentially expressed between JXS and WT (Table 1). In our study, the unigenes corresponding to *T5H*, *TAT*, *T10OH*, and *DBBT* were strongly upregulated, while taxoid 13-alpha-hydroxylase- (*T13OH*-) and 10-deacetyl baccatin III acetyltransferase- (*DBAT*-) encoding genes showed a significantly decreased transcript abundance in JXS. Among them, *T5H* and *T10OH* involved in hydroxylation steps were more highly expressed in JXS, which were 11.5 and 6.5 times of WT, respectively. These results indicated that JXS had more active hydroxylation and acylation reactions, except for the steps regulated by *T13OH* and *DBAT*. Moreover, there is no difference between WT and JXS for *DBTNBT*. The unigenes encoded for *T7OH*, *T2OH*, *TS*, and *BAPT* showed 0.6-fold, 0.7-fold, 0.4-fold, and 1.6-fold upregulation in JXS, respectively. However, any difference in these genes is not significant.

### 3.5. DEGs Involved in the Plant Hormone Signal Transduction Pathway

The plant hormone signal transduction pathway is also considered to be crucial in affecting paclitaxel biosynthesis. A large number of DEGs were found in the plant hormone signal transduction pathway between JXS and WT, which were mainly enriched in JA, GAs, auxin, and ET signal transduction. Our RNA-seq data showed that coronatine-insensitive protein 1- (COI1-) and jasmonate ZIM domain-containing protein- (JAZ-) encoding genes had significantly increased transcript abundance, while the gene encoding for MYC2 showed 1.9-fold upregulation in the JA signaling pathway of JXS. In the GA signaling pathway, DELLA-encoding genes showed a similar trend with the JAZ gene, while unigenes encoding for GID1 were downregulated. Moreover, JXS showed strongly upregulated genes corresponding to the auxin response factor (ARF), SAUR, and GH3 in the auxin signaling pathway and PRB1 in the SA signaling pathway, and only LAX (in the auxin signaling pathway) had a trend opposite to the others. For the ET signaling pathway, the unigene encoding for the ERF1 TF was significantly upregulated in JXS. Most of these DEGs were associated with cell growth and defense response (Table 2).

Linolenic acid metabolism, tryptophan metabolism, cysteine and methionine metabolism, and diterpenoid biosynthesis pathways were in response to the biosynthesis of JA, auxin, ET, and GA, respectively. Our results showed that two unigenes in the biosynthesis of JA, linoleate 9S-lipoxygenase (LOX), and oxophytodienoate reductase (OPR) showed increased transcript abundance in JXS. Similarly, genes encoding for GA3 in the biosynthesis of GA and amidase domain-containing protein (AMDD) and aldehyde dehydrogenase (aldA) in the auxin biosynthesis pathway were significantly upregulated. These results implied a high expression of JA, auxin, and GA in the leaves of JXS. In contrast, in the biosynthesis of ET, a series of genes corresponding to the met family, including adenosylhomocysteinase (AHC1) and 1-aminocyclopropane-1-carboxylate oxidase (ACO), displayed decreased transcript abundance, which implied a lower ET level in JXS (Table 2).

### 3.6. Regulation of the Expression of TFs between WT and JXS

TFs can activate the coexpression of multiple genes in secondary metabolic pathways, thus effectively regulating secondary metabolite production. Our transcriptome data showed that 975 unigenes were annotated to encode putative TFs (Table S6). These TFs were largely represented by families such as the ERF superfamily, MYB superfamily, bHLH superfamily, and WRKY superfamily. The DEGs encoding for TFs between JXS and WT were mainly involved in the regulation of the secondary metabolites and the defense response. Among them, the expression levels of 18 genes encoding for TFs showed a higher transcript abundance, including the members of GRAS, G2, LBD, bHLH, MYB, B3, NAC, and GeBP domain-containing TFs, while 3 genes corresponding to zinc finger proteins (C2H2 and C3H) displayed dramatic decreases in expression in JXS. Moreover, 5 out of 8 genes encoding for ERFs showed a higher transcript abundance, and the remaining showed lower expression levels in JXS. Abundant unigenes encoding putative TFs between JXS and WT showed that transcription regulation played a key role in taxol biosynthesis and the defense response network (Table S6).

### 3.7. Validation of DEGs by qPCR

The expression levels of the DEGs obtained by transcriptomic sequencing were verified by quantitative RT-PCR of 12 randomly selected DEGs involved in taxol biosynthesis, plant hormone biosynthesis and signal transduction, and TFs. Our results showed that the relative expression levels of 10 out of 12 tested genes were similar to those of transcriptomic sequencing, while the expressions of Unigene0087698 (GID1) and Unigene0061245 (DELLA) in qPCR assays had opposite expression patterns from RNA-seq analysis. It is notable that genes encoding for GID1 and DELLA are involved in the GA signaling pathway.

## 4. Discussion

**Figure 5:**
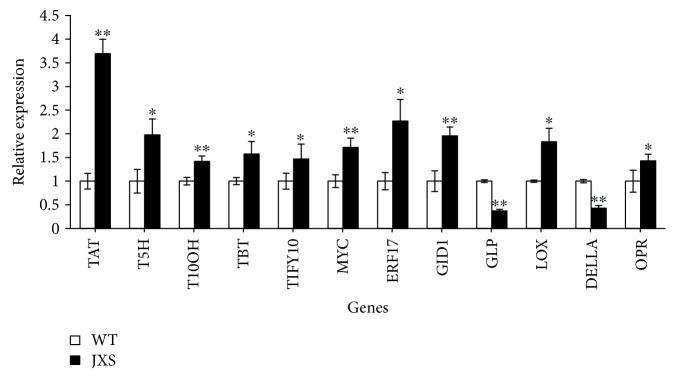
Expression analysis of 12 randomly selected genes as determined by qPCR.

*T. mairei* is a valuable source of paclitaxel [26–29]. However, quite low taxol contents have been reported in the reproducible tissues of *T. mairei*, such as branches and leaves, which are only 0.0013–0.0018% and 0.0004–0.0014%, respectively [30]. The values in the barks and roots are as high as 0.0241% and 0.0353%, respectively [31, 32]. However, obtaining taxol from barks or roots may cause permanent damage, resulting in the destruction of *Taxus* plants. Therefore, it is necessary to explore effective methods to separate paclitaxel and ensure that resources of *Taxus* are not destroyed. In the present study, a cultivar with yellow arils was selected from a population of *T. mairei* and was named JXS. Interestingly, the content of taxol in the leaves of JXS is about 3 times higher than that of WT. These results provide a new perspective for the extraction of taxol and related precursors from reproducible tissues. However, with limited knowledge of the regulation of taxol biosynthesis, it is necessary to elucidate the specific biosynthesis mechanism in JXS. Therefore, RNA-seq analysis was performed on the leaves of JXS and WT. A large number of unigenes were detected to have different transcriptional levels between WT and JXS, and the expression levels of most randomly selected genes in qPCR assays showed a similar trend, which demonstrated the reliability of *Taxus* transcriptome data accordingly (Figure 5).

**Figure 6:**
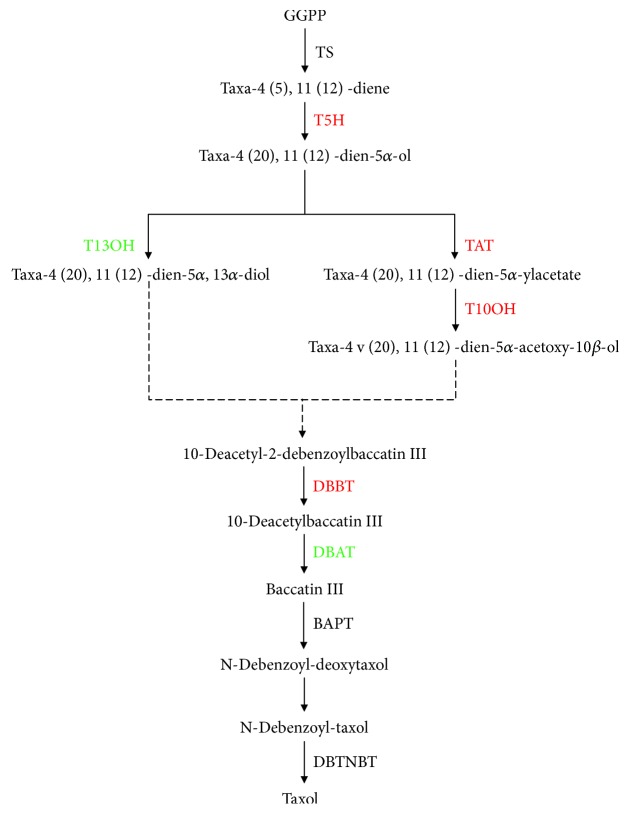
DEGs assigned to the taxol biosynthesis pathway. Red letters indicate the upregulation of gene expression; green letters indicate the downregulation of gene expression.

Studies have confirmed that the genotype differences of taxol accumulation are mainly determined by the expression of DEGs in the taxol biosynthesis pathway [33–35]. Therefore, known genes involved in the taxol biosynthesis pathway were identified by RNA-seq analysis to elucidate the related molecular mechanisms in the present study (Figure 6). Previous studies have proved that the acetylation step catalyzed by *TAT* is the rate-limiting step for the downstream hydroxylation reactions [36]. *DBBT* is a key enzyme involved in the downstream formation of 10-deacetyl-2-debenzoylbaccatin III (10-DAB III), which is one of the most important precursors for taxol synthesis [34]. In our results, genes encoding for *TAT* and *DBBT* displayed a significant increase in JXS. Moreover, genes encoding for key enzymes *T5H* and *T10OH* in the hydroxylation steps, which may be affected by the *TAT* catalytic step, were also highly expressed in JXS, indicating a series of more active hydroxylation reactions in the taxol biosynthesis pathway. These combined results implied a large amount of 10-DABIII accumulated in the needles of JXS, which may be the main reason for the high taxol content. However, the unigene encoding for *DBAT*, which converts 10-DAB III to baccatin III as the last diterpenoid intermediate before taxol [37], showed a lower expression in JXS, implying a lower conversion of 10-DAB III to taxol [38]. A study has proved the limiting effect conducted by *DBAT* in the taxol biosynthesis pathway [39]. The large accumulation of 10DABIII could explain the downregulation of *DBAT* and high accumulation of taxol in the leaves of JXS. A similar phenomenon was also observed in the leaves of a new *Taxus yunnanensis* cultivar with higher taxol accumulation [4]. Further investigation into the regulation of these genes is thus required to further understand paclitaxel biosynthesis. The expressions of these DEGs can further provide reasonable explanations for the changes in the content of paclitaxel between WT and JXS.

**Figure 7:**
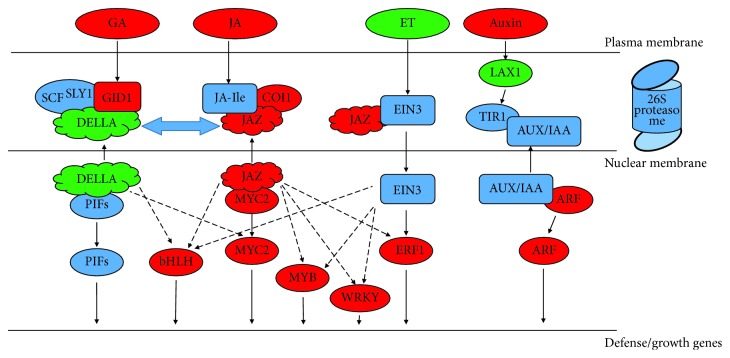
DEGs assigned to plant hormone signal transduction. Red letters indicate the upregulation of gene expression; green letters indicate the downregulation of gene expression.

Increasing evidence shows that hormone signal transduction pathways have important regulatory effects on the biosynthesis of secondary metabolites [40, 41]. The JA signaling pathway has been found to induce taxol biosynthesis in *Taxus* cells [42]. In this study, a large number of JA-related DEGs were identified, suggesting variations in JA biosynthesis and signaling between JXS and WT. The activation of JA signaling was derived from the binding of COI1 to JAZ, which marks the complex for degradation by the 26S proteasome in the presence of JA-Ile and frees MYC2, which in turn helps regulate the expression of a series of JA-inducible genes [43, 44]. The increased transcript abundances of COI1, JAZ, and MYC2 genes suggested a more activated JA signaling pathway in JXS, which may provide an explanation for the expression of downstream DEGs related to taxol biosynthesis. Moreover, the upregulation of genes *LOX* and *OPR* (*α*-linolenic acid pathway) in JXS further suggested that the activation of the JA biosynthesis process may lead to a higher JA level, which indicates the importance of the JA signaling transduction pathway (Figure 7).

The JA signal pathway has also been reported to crosstalk with other signal transduction pathways in the biosynthesis of secondary metabolites, such as GA, auxin, and ET signaling [45–48]. DELLA protein, which has a similar role with JAZ, participates in the activation of GA-responsive genes by interacting with GID1 for degradation by the 26S proteasome in the presence of F-box SLY1. The upregulation of the GID1 gene and low expression of the DELLA gene in qPCR assays (Figure 5), which showed an opposite trend from RNA-seq analysis, indicated that JXS has a more activated GA signaling pathway that interacts with the JA pathway in taxol biosynthesis. It has been shown that DELLA interacts with JAZ [49], participating in the release of TFs such as PIF and MYC2 [47] to balance the JA and GA signaling pathways. Therefore, mutual promotion between JA and GA in JXS could be a means for regulating development and the defense response. Similarly, a more activated auxin signaling pathway was also observed in JXS through the high expression levels of a series of key genes encoding for ARF, SAUR, and GH3, but not LAX. The synergistic effect between auxin and JA as well as GA and JA in JXS further indicates a specific regulatory pattern between plant development and defense in JXS. Given the complexity of multiple signal interaction networks, it is still necessary to conduct independent validations to accurately measure the expression levels of genes of interest. Moreover, the decrease of a series of genes related to ET biosynthesis (cysteine and methionine pathway) implied a decrease in the ET level in JXS, which indicated that ET may be involved in negatively regulating taxol biosynthesis. The increased transcript abundance of the ERF1 gene in JXS may be due to the regulation of JAZ [45]. Our results were basically consistent with those of Zhang and Wu [50]. However, Sun et al. [19] indicated that JA and ET regulate the increase of paclitaxel content through a synergistic effect mediated by MeJA. The reason could be the different response times and ratios of a series of endogenous hormones (Figure 7).

TFs play a key role in the regulation of secondary metabolite production [51]. Many studies have reported a series of TFs that can increase the expression of paclitaxel synthesis genes [52–54]. Our results showed a differential expression of several TF-encoding genes, including ERF, bHLH, MYB, and WRKY, between JXS and WT, which may be involved in the regulation of genes in the taxol biosynthesis pathway. Lenka et al. reported that TcJAMYC2 had a negative regulatory role on the expression of genes encoding for *TS*, *T5H*, *DBAT*, *DBBT*, *PAM*, *BAPT*, and *DBTNBT*. However, it has also been reported that the overexpression of TcMYC2a could increase the expression of *TS*, *T5H*, *DBTNBT*, *T13H*, and *TAT* [55]. As the fatal point of the entire JA signaling pathway [56], the MYC2 was 1.9-fold upregulated in JXS, which showed a similar expression pattern with *T5H*, *TAT*, *T10OH*, and *DBBT*, while opposite to *T13H* and *DBAT*, indicating a specific regulatory role on the taxol biosynthesis. Moreover, Li et al. [57] reported the positive regulation of TcWRKY1 on its target gene *DBAT*, which was inconsistent with our study. The reason could be attributed to the different regulation patterns of WRKY in different *Taxus* species. Interestingly, many ERFs, such as ERF114, ERF018, and ERF016, were up- or downregulated in the needles of JXS, which may act as negative or positive regulators on the genes in the taxol biosynthesis pathway. Similar cases were also reported by He et al. [4] and Zhang et al. [58], who considered that the dual regulations of ERFs as repressors and activators were mainly mediated by hormonal signal transduction. Therefore, one major regulatory mechanism of taxol production in JXS is via the control of the expression of TFs, which can probably be ascribed to the crosstalk between JA and other hormonal signaling. Moreover, all of the other TFs that were differentially expressed in the transcriptome profiles between JXS and WT are involved in cell growth and the defense response, such as NAC, LBD, and zinc fingers. These results indicated that many genes encoding TFs may mediate the regulation of plant growth and development, as well as biotic and abiotic stress responses, thus regulating the production or activity of taxol biosynthetic enzymes directly or indirectly. Therefore, characterization of the DEGs that encode TFs might shed light on the regulation of taxol biosynthesis in *Taxus* species.

## 5. Conclusion

The taxol content in the needles of a new cultivar of *T. mairei* (JXS) is about 3-fold higher than in WT. Transcriptome profiling was conducted for the first time to illustrate the regulatory mechanism of taxol biosynthesis in the leaves of JXS and WT. The differentially expressed genes encoding key enzymes in the taxol biosynthesis pathway explain the high content of taxol in the needles of JXS. The variations of plant hormone-related genes, including hormone signal transduction and hormone biosynthesis genes, might be responsible for enhancement in the expressions of paclitaxel biosynthesis genes in JXS. Moreover, DEGs encoding for transcriptional factors were detected, which helps us understand the regulatory patterns and molecular mechanisms of hormone-mediated taxol biosynthesis. In summary, these transcriptome data provide reasonable explanations for the variation of taxol content between WT and JXS.

## Figures and Tables

**Table 1:** Putative functional genes involved in taxol biosynthesis.

Unigene ID	Description	WT_rpkm	JXS_rpkm
Unigene0083800	Taxadiene synthase (TS)	16.093	6.672
Unigene0014731	Taxadiene 5-alpha hydroxylase (T5H)	0.084	0.965^∗^
Unigene0087496	Taxadienol acetyltransferase (TAT)	3.382	10.698^∗^
Unigene0084941	5-Alpha-taxadienol-10-beta-hydroxylase (T10OH)	3.488	22.619^∗^
Unigene0013222	Taxane 13-alpha-hydroxylase (T13OH)	5.965	2.830^∗^
Unigene0105070	2-Debenzoyl-7,13-diacetylbaccatin III-2-O-benzoyl transferase (DBBT)	0.001	0.528^∗^
Unigene0077022	10-Deacetylbaccatin III-10-O-acetyl transferase (DBAT)	1.451	0.323^∗^
Unigene0100486	Baccatin-aminophenylpropanoyl-13-O-transferase (BAPT)	9.193	14.470
Unigene0075463	Taxoid 7-beta-hydroxylase (T7OH)	10.510	6.692
Unigene0014173	3′-N-Debenzoyltaxol N-benzoyltransferase (DBTNBT)	30.895	32.159
Unigene0095993	Taxoid 2-alpha-hydroxylase (T2OH)	10.458	7.108

**Table 2:** Putative genes involved in plant hormone biosynthesis and signal transduction pathways.

Unigene ID	Annotation	Fold change	*p* value	FDR
*Putative genes involved in plant hormone signal transduction*				
Unigene0097028	Coronatine-insensitive protein 1 (COI1)	66.760	3.285*E* − 11	4.582*E* − 09
Unigene0075828	Protein TIFY 10B (TIF10B)	4.310	3.392*E* − 05	1.433*E* − 03
Unigene0076962	Protein TIFY 9 (TIFY9)	4.684	1.116*E* − 04	4.033*E* − 03
Unigene0000384	PREDICTED: transcription factor MYC2 isoform X1 (BHLH82)	1.879	1.646*E* − 03	3.779*E* − 02
Unigene0087698	GLP1 GID1-like protein (GID1C)	0.232	3.641*E* − 05	1.524*E* − 03
Unigene0002231	GLP1 GID1-like protein (GID1A)	0.277	4.347*E* − 04	1.264*E* − 02
Unigene0061245	DELLA protein RGL2 (RGL2)	13.737	2.024*E* − 05	9.064*E* − 04
Unigene0093951	Ethylene-responsive transcription factor 1-like protein (ERF1)	2.927	1.174*E* − 03	2.873*E* − 02
Unigene0065363	Auxin response factor 12 (ARF12)	64.041	9.467*E* − 11	1.236*E* − 08
Unigene0033519	Auxin-responsive protein SAUR71 (SAUR71)	2.576	1.876*E* − 03	4.179*E* − 02
Unigene0000975	Indole-3-acetic acid-amido synthetase GH3.6 (GH3.6)	4.998	4.095*E* − 10	4.745*E* − 08
Unigene0085639	Pathogenesis-related protein 1C (PRB1)	3.380	7.595*E* − 05	2.876*E* − 03
Unigene0069610	Auxin transporter-like protein 1 (LAX1)	0.276	3.603*E* − 07	2.470*E* − 05

*Putative genes involved in plant hormone biosynthesis*				
Unigene0101434	Linoleate 9S-lipoxygenase (LOX1.1)	11.569	7.045*E* − 16	1.710*E* − 13
Unigene0054914	12-Oxophytodienoate reductase 1 (OPR1)	7.557	2.193*E* − 04	7.186*E* − 03
Unigene0098962	Ent-kaurene oxidase-like protein 1 (CYP701A7)	13.743	3.006*E* − 20	1.043*E* − 17
Unigene0107104	Cystathionine gamma-synthase 1, chloroplastic (CGS1)	0.002	5.143*E* − 04	1.455*E* − 02
Unigene0046234	1-Aminocyclopropane-1-carboxylate oxidase 1 (ACO1)	0.005	3.799*E* − 31	3.113*E* − 28
Unigene0104890	S-Adenosylmethionine synthase (metK)	0.001	5.879*E* − 14	1.139*E* − 11
Unigene0020386	Adenosylhomocysteinase A (AHC1)	0.001	5.092*E* − 28	3.182*E* − 25
Unigene0038147	5-Methyltetrahydropteroyltriglutamate-homocysteine methyltransferase (met6)	0.002	4.686*E* − 05	1.898*E* − 03
Unigene0039863	5-Methyltetrahydropteroyltriglutamate-homocysteine methyltransferase (met26)	0.002	2.037*E* − 03	4.447*E* − 02
Unigene0113472	Amidase domain-containing protein (AMDD)	9.589	8.688*E* − 09	8.028*E* − 07
Unigene0096072	Aldehyde dehydrogenase (aldA)	4.430	1.296*E* − 08	1.164*E* − 06

## Data Availability

The data used to support the findings of this study are included within the supplementary information files. The rest of the transcriptome sequencing data are currently under embargo while the research findings are commercialized. Requests for data, 6/12 months after publication of this article, will be considered by the corresponding authors.

## Supplementary Materials

Supplementary 1 Table S1: primers used in qRT-PCR for the validation of differentially expressed genes.

Supplementary 2 Table S2: sequences of unigenes in WT and JXS.

Supplementary 3 Table S3: four database annotation statistics.

Supplementary 4 Table S4: annotated unigenes were assigned to KEGG pathways.

Supplementary 5 Table S5: putative 109 genes involved in taxol biosynthesis.

Supplementary 6 Table S6: annotation to encode putative TFs.
